# Accumulation dynamics of ARGONAUTE proteins during meiosis in *Arabidopsis*

**DOI:** 10.1007/s00497-021-00434-z

**Published:** 2021-11-23

**Authors:** Cecilia Oliver, German Martinez

**Affiliations:** Department of Plant Biology, Uppsala BioCenter, Swedish University of Agricultural, Sciences and Linnean Center for Plant Biology, Uppsala, Sweden

**Keywords:** Meiosis, RNA silencing, Argonaute, Small RNAs, Pollen, Epigenetics

## Abstract

Meiosis is a specialized cell division that is key for reproduction and genetic diversity in sexually reproducing plants. Recently, different RNA silencing pathways have been proposed to carry a specific activity during meiosis, but the pathways involved during this process remain unclear. Here, we explored the subcellular localization of different ARGONAUTE (AGO) proteins, the main effectors of RNA silencing, during male meiosis in *Arabidopsis thaliana* using immunolocalizations with commercially available antibodies. We detected the presence of AGO proteins associated with posttranscriptional gene silencing (AGO1, 2, and 5) in the cytoplasm and the nucleus, while AGOs associated with transcriptional gene silencing (AGO4 and 9) localized exclusively in the nucleus. These results indicate that the localization of different AGOs correlates with their predicted roles at the transcriptional and posttranscriptional levels and provide an overview of their timing and potential role during meiosis.

**Supplementary Information:**

The online version contains supplementary material available at 10.1007/s00497-021-00434-z.

## Introduction

Meiosis is a special type of cell division key for the production of gametes and the reshuffling of the genetic information during sexual reproduction (Bolcun-Filas et al. 2018). During meiosis, one round of DNA synthesis is followed by two rounds of cell division, segregating homologous chromosomes during the first division and sister chromatids at the second division (Marston et al. 2004; Mercier et al. 2015). The mechanisms regulating meiosis have been widely studied at the cellular, genetic, and molecular levels in a variety of organisms (Marston et al. 2004). In plants, more than 90 genes have been identified comprising different meiotic processes that include double-strand break (DSB) formation, chromosome segregation, or meiotic recombination (Huang et al. 2019). Intriguingly, several species across the tree of life use RNA interference/RNA silencing to ensure genome stability and regulate gene expression during gametogenesis (Goh et al. 2015; Hall et al. 2003; Hammond et al. 2013; He et al. 2009; Holmes et al. 2007; Lepere et al. 2009; Modzelewski et al. 2012; Wang et al. 2015). In plants, different RNA silencing pathways are active during meiosis (Huang et al. 2019, 2020 ; Yelina et al. 2015). For example, in maize and rice, the miRNA pathway regulates the production of 21-nt and 24-nt phased siRNAs that accumulate and regulate gene expression in premeiotic and meiotic stages (Dukowic-Schulze et al. 2016; Komiya et al. 2014; Zhai et al. 2015). In *Arabidopsis,* the miRNA pathway is crucial for the development of male gametes (Borges et al. 2011) and it is known to affect chromatin condensation and the number of chiasmata (Oliver et al. 2016, 2017). The other main RNA silencing pathway in plants, the RNA-directed DNA methylation (RdDM) pathway, affects chromatin condensation, the number of chiasmata and chromosome segregation (Oliver et al. 2016, 2017) and protects euchromatic regions from meiotic recombination in *Arabidopsis* (Yelina et al. 2015). In *Arabidopsis*, meiocyte-specific sRNAs between 23–24 nts are positively correlated with genes that have a meiocyte-preferential expression pattern (Huang et al. 2019), which could correlate with the observed role of DNA methylation in the regulation of gene expression in meiocytes (Walker et al. 2018). Furthermore, in maize ARGONAUTE (AGO) proteins associated with RdDM activity have been linked to apomixis-like phenotypes (Singh et al. 2011). Additionally, in *Arabidopsis,* a non-canonical RNA silencing pathway containing members from different RNA silencing pathways plays a role in DSB repair (Wei et al. 2012). AGO proteins are the effectors of the different RNA silencing pathways and have dedicated members that act at the posttranscriptional or transcriptional levels. Nevertheless, their dynamism and subcellular localization during meiosis in plants are unknown. Here, we analyzed the subcellular localization of the main AGO proteins in *Arabidopsis* during different meiotic stages, shedding light onto their potential roles during this process.

## Materials and methods

### Plant material

Plants used for immunolocalization analysis were grown in a phytotron under long-day conditions (16-h light/8-h dark photoperiod), at 24–25 °C and 45% relative humidity.

### Bioinformatic analysis

sRNA data was downloaded from the SRA repository project number PRJNA510650 (Huang et al. 2019). sRNA alignments were performed using bowtie (Langmead et al. 2009) with the following parameters –t –v2 that allows two mismatches to the alignments. Alignment files were subsequently analyzed in Galaxy (Afgan et al. 2018). For sRNA categorization as miRNAs, sRNA libraries were aligned to individual indexes generated for each genomic category and compared total sRNAs mapping to the TAIR10 chromosome sequences. The miRbase version 22.1 (https://www.mirbase.org/) was used for miRNA alignments (Kozomara et al. 2019). Transcriptomic data correspond to the CATMA arrays data from GEO accessions GSE10229 and GSE13000 (Libeau et al. 2011) and RNA sequencing data from the GEO accession GSE86583 (Walker et al. 2018). CATMA array data were extracted using the CATdb database (http://urgv.evry.inra.fr/cgi-bin/projects/CATdb/catdb_index.pl) were normalized data were extracted for both GSE10229 (http://urgv.evry.inra.fr/cgi-bin/projects/CATdb/consult_expce.pl?experiment_id=195) and GSE13000 (http://urgv.evry.inra.fr/cgi-bin/projects/CATdb/consult_expce.pl?experiment_id=46). RNA sequencing data were downloaded, adapter trimmed, and filtered by length and quality using Trim Galore! (https://www.bioinformatics.babraham.ac.uk/projects/trim_galore/). For gene expression analysis, paired reads were aligned to the Arabidopsis TAIR10 genome using bowtie2 (Langmead et al. 2012) using default parameters. Count reads were obtained using HTSeq-COUNTS (Anders et al. 2015) with the following parameters: –mode union –stranded no –minequal 10 and –nonunique none. The obtained count tables were used in DESeq2 (Love et al. 2014) to infer significant expression with fit type set to parametric. All these tools were used on the Galaxy platform (Afgan et al. 2018).

### Cytology

Immunolocalization on meiotic nuclei was carried out by squash technique as was previously described by Manzanero et al. (2000) with some modifications (Oliver et al. 2013). Two bioreplicates constituted by young flower buds from five different plants were analyzed. Young flower buds were fixed for 20 min in freshly prepared 4% (w/v) paraformaldehyde, 0.1% (v/v) Triton X-100 in phosphate-buffered saline (PBS, pH 7.3). Flower buds were then washed at room temperature for 30 min in PBS that was changed twice. Buffer was removed before incubation at 37 °C during 20–40 min with an enzyme mixture of 1% pectinase, 1% cellulase, and 1% cytohelicase (w/v) (Sigma), dissolved in PBS. Buds, immersed in a small volume of PBS, were transferred to slides with a Pasteur pipette, macerated with a needle, and squashed between a glass slide and cover slip. After freezing in liquid nitrogen, the cover slips were removed and the slides were transferred immediately into PBS. Prior to immunostaining experiments, the slides were washed twice in PBS, 0.1% (v/v) Triton X-100 for 5 min each. To avoid non-specific antibody binding, slides were incubated for 30 min in PBS with 1% BSA (w/v) and 0.1% Triton X-100 at room temperature. The incubation with the primary antibody was carried out in a humidified chamber. The primary antibodies used were rabbit anti-AGO1 (1:200 AS09 527), -AGO2 (1:100, AS13 2682), -AGO5 (1:100, AS10 671), -AGO4 (1:100, AS09 617), -AGO6 (1:50, AS10 672), -AGO9 (1:100, AS10 673), and -AGO10 (1:50, AS15 3071) antibodies from Agrisera. All the primary antibodies were diluted in PBS, 1% BSA, 0.1% Triton X-100. After overnight incubation at 4ºC and washing for 15 min in PBS with 0.1% Triton X-100, the slides were incubated for 1 h at room temperature with goat anti-rabbit IgG H&L Alexa Fluor 568 conjugated (1:200; ab175471; Abcam) diluted in 1% BSA, 0.1% Triton X-100 in PBS. Slides were then washed in PBS, 0.1% Triton X-100, before they were stained the DAPI, 1 μg/ml during 20–30 min and finally mounting with antifading medium (0.2% n-propyl Gallete, 0.1% DMSO, 90% glycerol in PBS). Fluorescent signals were observed using an epifluorescence microscope Zeiss AxioScope A1. Images were captured with AxioCam ICc5 camera and were analyzed and processed with ImageJ and Affinity Photo software. The number of cells observed for each meiotic stage is shown in Supplementary Table 3. (Manzanero et al. 2000; Oliver et al. 2013).

## Results and discussion

**Fig. 1:**
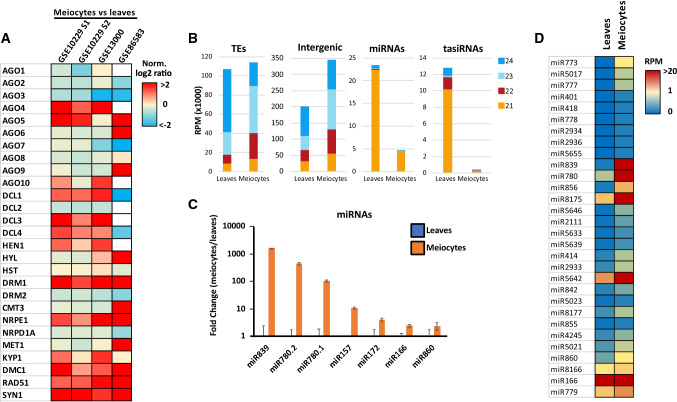
Analysis of the expression in meiocytes of different RNA silencing and epigenetic pathways components and analysis of sRNA and miRNA accumulation in meiocytes. **A** Heat map of the expression values of RNA silencing and epigenetic pathways components in meiocyte microarray and RNA sequencing experiments. Expression values are represented as the normalized log2 ratio of the comparison meiocyte/leaves. Expression values of genes with known expression in meiocytes (DMC1, RAD51, and SYN1) are shown as a control of the meiocyte origin of the datasets. Datasets analyzed correspond to the data from Libeau et al. (2011) (CATMA microarrays; GSE10229 and GSE13000) and Walker et al. (2018) (RNA sequencing; GSE86583) ** B**. Global accumulation of sRNAs from 21- to 24-nt in length from public datasets (Huang et al 2019; PRJNA510650) derived from TEs, intergenic regions, miRNAs and tasiRNAs in leaves and meiocytes. Accumulation values are expressed in thousand reads per million (RPM × 1000). ** C** Accumulation values of selected miRNAs enriched in meiocyte sRNA libraries. Accumulation is expressed as the fold change of the ratio between meiocytes and leaves of the accumulation value for each miRNA family in reads per million. Enrichment was considered only for miRNAs accumulating more than twofold in meiocytes and with a *p*-value < 0.05. ** D** Heat map of the accumulation values of all the miRNA families enriched in meiocytes. Enrichment was considered only for miRNAs accumulating more than twofold in meiocytes compared to leaves Accumulation is expressed in reads per million

To discern the level of expression of RNA silencing components in *Arabidopsis* meiocytes, we analyzed their relative expression in publicly available microarray (Libeau, et al. 2011) and RNA sequencing datasets (Walker et al. 2018) (Fig. 1 and Supplementary Methods). Overall, several components from the RNA silencing pathways were preferentially expressed in meiocytes compared to somatic tissues in at least three of the four datasets analyzed (Fig. 1a). These included the AGO proteins AGO4 and 5, the DICER-LIKE (DCL) proteins DCL1, 3, and 4, or the sRNA methyltransferase HEN1, member of the miRNA pathway. Interestingly, we also detected enriched expression of the methyltransferase DRM1 and the RNA polymerase V (Pol V) (Fig. 1a). Overall, despite differences between the datasets analyzed, our analysis indicated that different PTGS (AGO5, HYL, DCL1, and DCL4) and TGS (AGO4, DCL3, and Pol V) pathways might be especially active during meiosis. Previous analysis has shown that transposable element (TE)-derived sRNAs accumulate to relatively high levels in meiocytes (Huang et al. 2019) and that certain miRNAs like miR845 are active before the pollen microspore stage (Borges et al. 2018). To understand sRNA accumulation during meiosis, we analyzed their categorization in publicly available sRNA sequencing data from purified meiocytes (Huang, et al. 2019). Our analysis showed that, as previously reported, there is a global decrease of 24-nt and an increase in 22 and 23-nt total sRNAs (Supplementary Fig. 1A). Several categories showed an enrichment in meiocytes, including TEs, intergenic regions, and several functional non-coding RNAs (specially rRNAs and tRNAs; Supplementary Fig. 1B). Other classes of sRNAs such as miRNAs and tasiRNAs were slightly depleted from meiocytes (Supplementary Fig. 1B). In the case of TEs and intergenic regions, the enrichment was observed specially for sRNAs of 21-, 22-, and 23-nts in length, while miRNAs and tasiRNAs lost a majority of 21-nt sRNAs (Fig. 1b). Despite miRNAs were not enriched in meiocytes compared to leaves (Supplementary Fig. 1a and Fig. 1b), several miRNA families were strongly upregulated in meiocytes including miR839, miR780.2, miR780.1, miR157, miR172, miR166, and miR860 (Figs. 1c and d and Supplementary Table 2). Overall, we identified 30 miRNA families enriched more than twofold in meiocytes compared to leaves (Fig. 1d). Within these upregulated miRNA families, there were miRNA families regulating important transcription factors including APETALA2, HD-ZIP III, Squamosa, or ethylene response factors (regulated, respectively, by miR172, miR166, miR157, and miR839; Supplementary Fig. 1). miRNA activity in meiocytes might be connected to the regulation of the expression of transcription factors. For example, HD-ZIP transcription factors are absent from meiotic tissues in *Physcomitrella patens* but accumulate in premeiotic tissues (Yip et al. 2016). Additionally, transcription factors like Squamosa are needed for male and female meiosis (Unte et al. 2003). Accordingly, the role of certain miRNAs (like miR166, which regulates HD-ZIP transcription factors) could be to exclude expression of certain transcription factor families or to tightly regulate their expression in meiocytes (which might be the role of miR157 and the regulation of Squamosa transcription factors). In summary, our re-analysis of transcriptomic and sRNA sequencing analysis supported the notion that the RNA silencing machinery might have a meiocyte-specific activity.

**Fig. 2:**
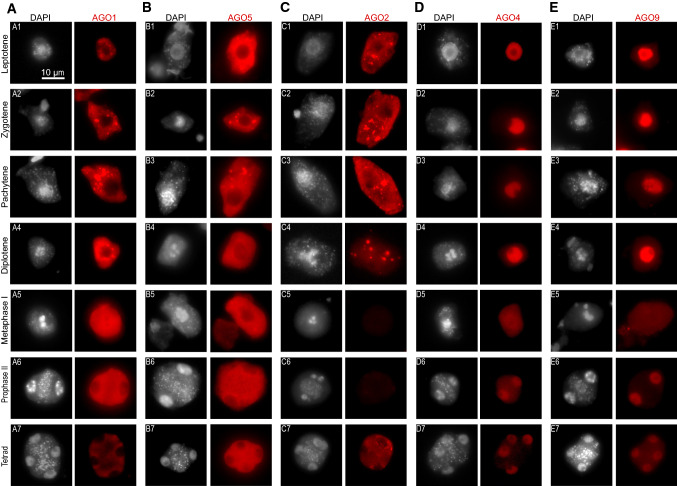
Immunolocalization of AGO1 (**a**), AGO5 (**b**), AGO2 (**c**), AGO4 (**d**), and AGO9 (**e**) at different representative meiotic stages in *Arabidopsis* meiocytes. Representative images of different meiotic stages in meiocytes as indicated in the left column: Leptotene (**a1**, **b1**, **c1**, **d1**, **e1**); Zygotene (**a2**, **b2**, **c2**, **d2**, **e2**); Pachytene (**a3**, **b3**, **c3**, **d3**, **e3**); Diplotene (**a4**, **b4**, **c4**, **d4**, **e4**); Diakinesis (B5), Metaphase I (**a5**, **c5**, **d5**, **e5**) Prophase II (**a6**, **b6**, **d6**, **e6**); Metaphase II (**c6**); Tetrad (**a7**, **b7**, **c7**, **d7**, **e7**). Immunostaining with antibodies is shown in red and counterstaining of nucleus and chromosomes with DAPI is shown in gray and indicated at the top of each image series. Bar indicates 10 µm and is shown as a reference of cell size

Although transcriptomic analyses are important to infer the activity of the different RNA silencing pathways in meiocytes, these analyses only provide a steady image of this tissue and ignore, for example, potential posttranscriptional regulation of mRNAs or the dynamism of protein accumulation during the whole meiosis progression. To understand the subcellular localization and dynamics of the different AGO proteins during meiosis, we performed immunolocalizations of the AGO proteins that had commercially available antibodies (Agrisera, AGO1, 2, 4, 5, 6, 9, and 10, Fig. 2 and Supplementary Fig. 2). Our approach allowed us to detect all AGOs but AGO6 and AGO10, which might be linked to a low accumulation of these proteins or a lack of sensitivity of our technical approach. In detail, AGO1 and its paralogs AGO2 and AGO5 displayed a similar localization and expression pattern during prophase I, when DSBs are formed and repaired, with only AGO2 showing spurious accumulation in the nucleus (Figs. 2a, b, and c, and Supplementary Figs. 2a, b, and c). The three proteins were located mainly in the cytoplasm, similar to their localization in somatic tissues (Bologna et al. 2018; Ye et al. 2012). From the leptotene to the diplotene stage these three AGO proteins formed cytoplasmic granules (Figs. 2a1, b1, and c1). In somatic tissues, cytoplasmic bodies are involved in the degradation and translation arrest of mRNAs (Maldonado-Bonilla 2014). In mammals, AGO proteins localize in P-bodies where they mediate the translational repression of their target mRNAs (Liu et al. 2005). The localization pattern observed for AGO1, 2, and 5 might indicate a similar role of RNA silencing in the posttranscriptional regulation of mRNAs, a process that is known to take place in other organisms like mammals (Yao et al. 2015). Additionally, this role might also be important for the posttranscriptional or translational repression of TEs (Kim et al. 2021), which are known to be active during meiosis (Yang et al. 2011) and regulated by miRNAs and easiRNAs which are potentially loaded in those AGO proteins (Borges et al. 2018; Creasey et al. 2014).

Despite the similarities between the accumulations during meiosis, AGO1, 2, and 5 showed differences in their dynamics during this division. For example, AGO5 displayed a similar pattern of subcellular localization to AGO1, although its localization at cytoplasmic bodies apparently disappeared at diplotene (Fig. 2b4). AGO5 and AGO1 load different populations of miRNAs and siRNAs in somatic tissues (Mi et al. 2008), so it is plausible that AGO5 delocalization from cytoplasmic bodies might be connected to the absence of their target mRNAs or their loaded miRNAs. On the other hand, AGO2 showed a dual localization in the cytoplasm and the nucleus (Figs. 2c1-4 and Supplementary Figs. 2C1-4) and was not detectable after metaphase I (Figs. 2c5-6 and Supplementary Fig. 2C5). Both its nucleocytoplasmic localization and timing of expression are in line with its known role in DSB repair, which takes place during the first meiotic stages (Oliver et al. 2014; Wei et al. 2012). It is plausible that the cytoplasmic localization of AGO2 is connected to the loading of DSB-associated sRNAs in the cytoplasm, similar to the loading of RdDM-associated 24-nt siRNAs in AGO4 (Ye et al. 2012). Nevertheless, AGO2 expression pattern was recapitulated in tetrads (Fig. 2c7 and Supplementary Fig. 2C6), indicating that it might serve other roles in parallel to its function in DSB repair during meiosis. In *Arabidopsis,* AGO2 accumulation is regulated by arginine methylation (Hu et al. 2019), which is a posttranslational modification associated with the regulation of the cell cycle (Raposo et al. 2018). It is plausible that the dynamic accumulation of AGO2 during meiosis might be associated with changes in its arginine methylation levels during meiosis progression, similar to the known regulatory role of this modification in the control of the *C.elegans* germline-specific AGO protein CSR-1A, which is an isoform that has specific accumulation during spermatogenesis (Nguyen et al. 2021).

On the other hand, the TGS/RdDM-associated AGO proteins, AGO4 and AGO9, were located in the nuclei during all meiotic stages (Figs. 2d and e and Supplementary Figs. 2D and E). Exceptionally, at metaphase I, when the nuclear envelope dissolves, both proteins showed a dispersed accumulation. This is in accordance with the known role of the RdDM pathway in regulating DNA methylation during meiosis (Walker, et al. 2018) and protecting against meiotic recombination in certain chromosomal regions such as pericentromeres (Underwood et al. 2018). Meiocytes have the lowest CHH methylation values of all the reproductive nuclei analyzed, but its activity is needed for the regulation of gene expression (Walker et al. 2018). We detected a low accumulation of AGO4 and 9 after metaphase I (Figs. 2d5-6 and e5-6 and Supplementary Figs. 2d5-6 and 2E5-6), which might partially cause this reduction in CHH methylation and might be connected to the low presence of 24-nt sRNAs in meiocyte sRNA libraries (Fig. 1b). The nuclear localization of AGO9 and AGO4 might explain their known roles in the dissolution of interlocks during meiosis and the mediation of appropriate chromosome segregation, respectively (Oliver et al. 2014, 2016). Several components of the PTGS and, specially, the TGS/RdDM pathway including RDR6, AGO9, and DRM1 and 2 control the specification of female gamete precursors, inhibiting the formation of ectopic megaspore mother cells (MMC) (Mendes et al. 2020; Olmedo-Monfil et al. 2010; Rodriguez-Leal et al. 2015). In developing ovules, AGO9 accumulates in cytoplasmic foci of the MMC companion cells and loads 24-nt sRNAs from TEs (Olmedo-Monfil et al. 2010). Our analysis indicates a divergent role of AGO9 during male meiosis, since we detected accumulation of this protein in the nucleus of male meiocytes, indicating a potentially active role in the establishment of DNA methylation. Interestingly, both the pollen mother cell (PMC) and the MMC experience analogous reorganization of chromatin which has been proposed to mediate the meiotic transcriptional program (She et al. 2015). Male meiotic sRNAs are important for the regulation of gene expression (Huang et al. 2019) and the inheritance of epigenetic states (Long et al. 2021). Indeed, DNA methylation levels analyzed through reporters indicate that non-CG methylation might be higher in tetrads than in microspores (Ingouff et al. 2017), suggesting that the RdDM activity could be dynamic through meiosis where it might play a transient role consistent with the regulation of the meiotic transcriptional program together with its known role in protecting against meiotic recombination (Underwood et al. 2018; Yelina et al. 2015).

In summary, our results (summarized in Supplementary Fig. 3) provide an overview of the subcellular localization, timing, and potential role of different RNA silencing pathways during meiosis. Furthermore, our work complements previous analysis that analyzed RNA silencing activity in meiocytes and opens the door for future molecular analysis of the specific role of AGO proteins during specific meiosis stages, which are technically challenging to purify for their analysis through high-throughput sequencing techniques at the moment.

### Author contribution statement

C.O and G.M. design the experiments and wrote the manuscript. C.O. performed the experiments and analyzed the data. G.M. analyzed the bioinformatic data.

## Supplementary Information

Below is the link to the electronic supplementary material.

Supplementary file1 (XLSX 38 KB)

Supplementary file2 (PDF 239 KB)

Supplementary file3 (PDF 1634 KB)

Supplementary file4 (PDF 78 KB)
