# Supplementary material for: Accumulation dynamics of ARGONAUTE proteins during meiosis in Arabidopsis
Source: Plant Reprod. 2021 Nov 23;35(2):153–60. doi: 10.1007/s00497-021-00434-z (PMC9110482; doi:10.1007/s00497-021-00434-z)

Supplementary Figure 1.

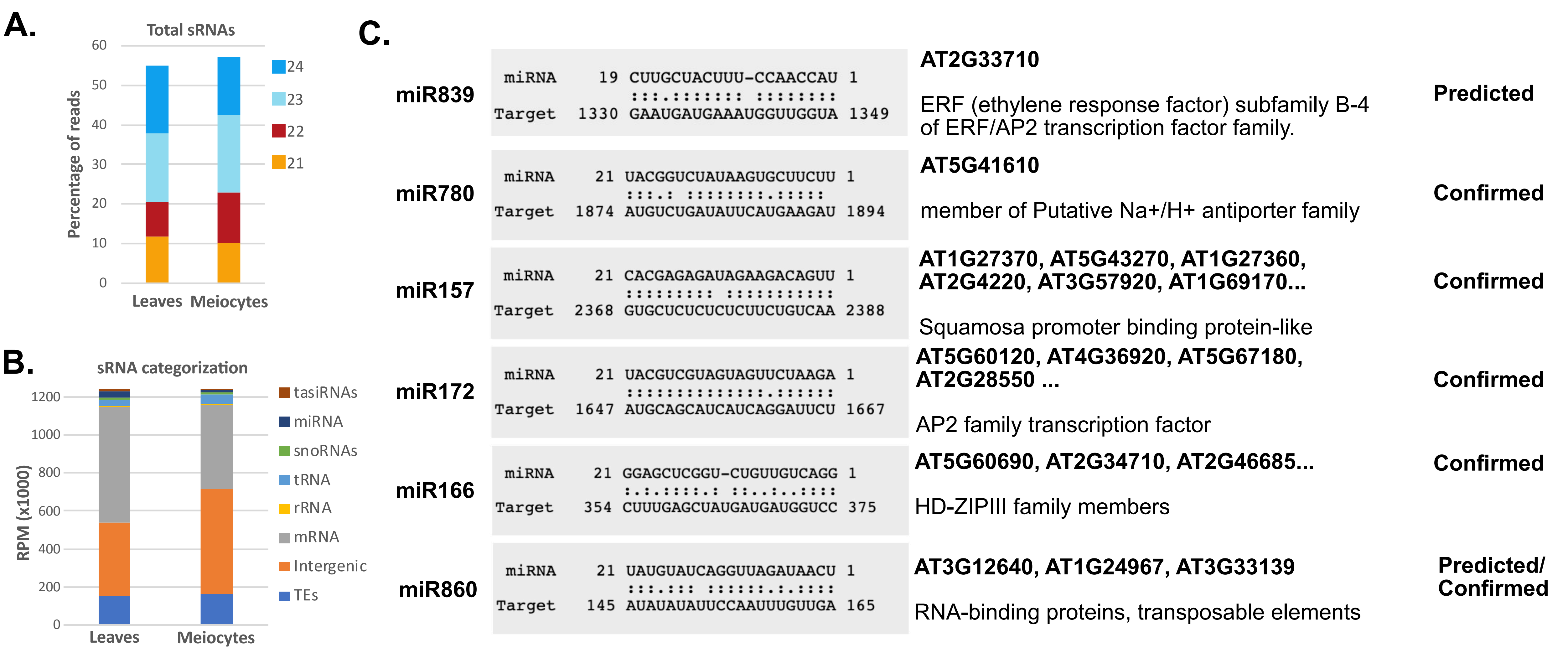

Supplement: Supplementary file 2 — Supplementary file2 (PDF 239 KB) [file 497_2021_434_MOESM2_ESM.pdf]
