# Supplementary figures and images for: Accumulation dynamics of ARGONAUTE proteins during meiosis in Arabidopsis
Source: Plant Reprod. 2021 Nov 23;35(2):153–60. doi: 10.1007/s00497-021-00434-z (PMC9110482; doi:10.1007/s00497-021-00434-z)

# Supplementary Figure 2.

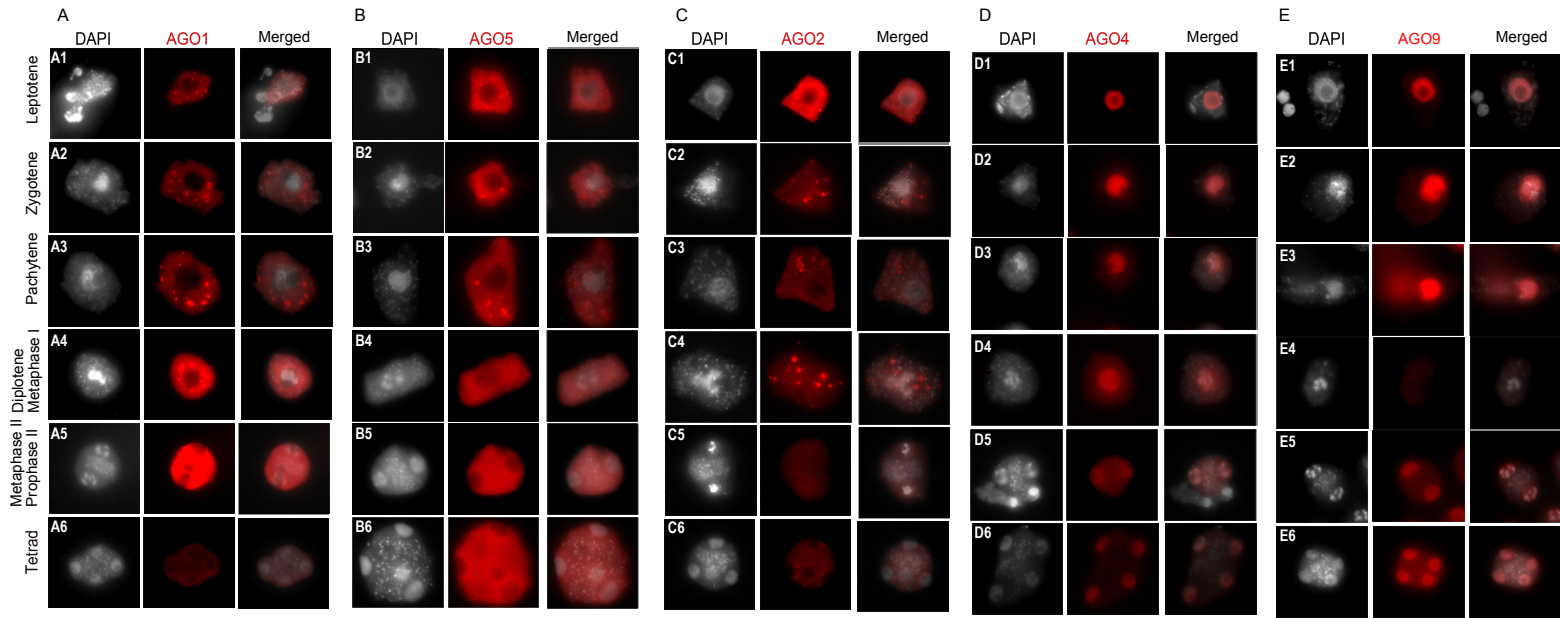

Supplement: Supplementary file 3 — Supplementary file3 (PDF 1634 KB) [file 497_2021_434_MOESM3_ESM.pdf]

# Supplementary Figure 3.

A.

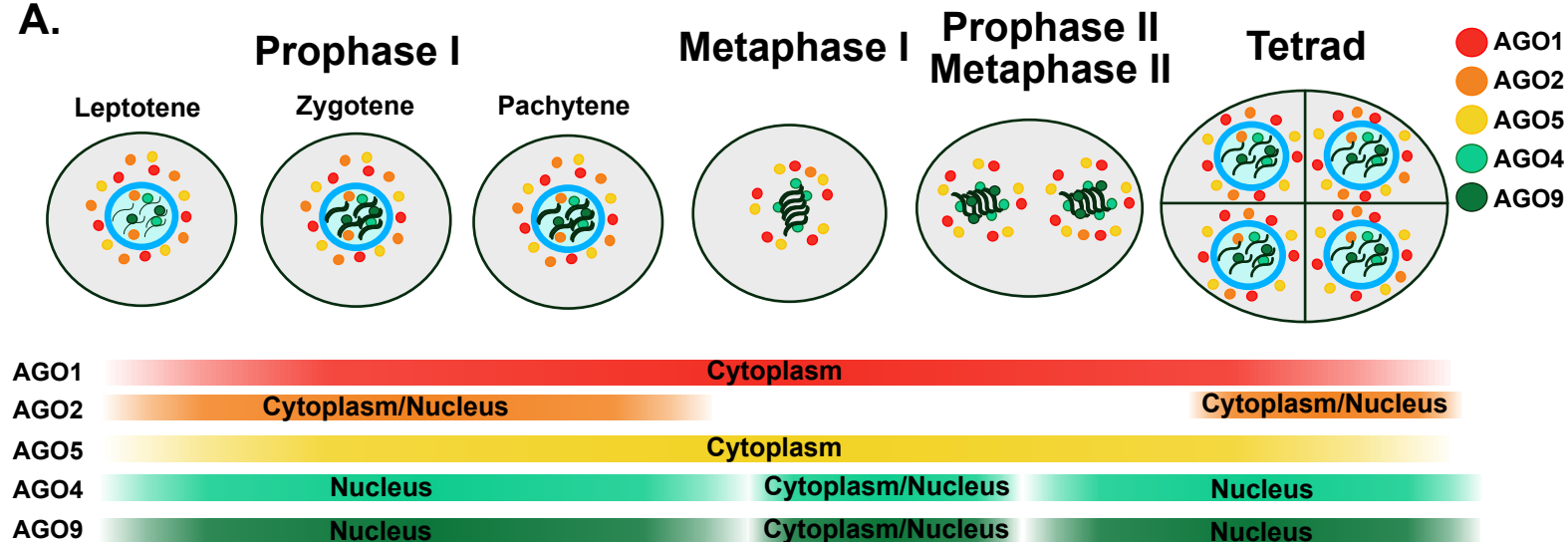

B.

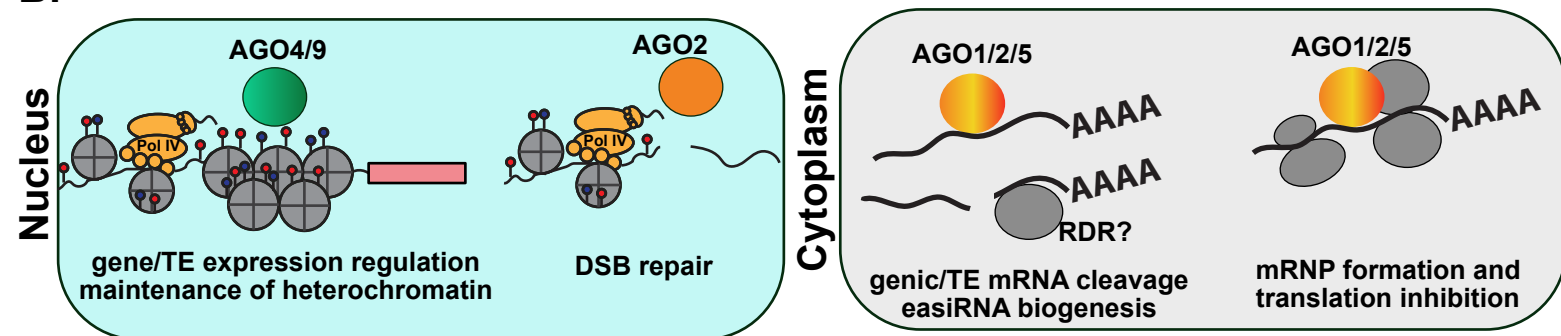

Supplement: Supplementary file 4 — Supplementary file4 (PDF 78 KB) [file 497_2021_434_MOESM4_ESM.pdf]
